# Intraoperative parathyroid hormone (PTH) testing in patients with primary hyperparathyroidism and PTH levels in the normal range

**DOI:** 10.1186/s12893-018-0459-3

**Published:** 2019-04-24

**Authors:** Fabio Medas, Enrico Erdas, Giulia Loi, Francesco Podda, Lucia Barca, Giuseppe Pisano, Pietro Giorgio Calò

**Affiliations:** 1 Department of Surgical Sciences, University of Cagliari, Cagliari, Italy; 2 Center for the Study of Liver Diseases, Department of Medical Sciences “M. Aresu”, University of Cagliari, Cagliari, Italy

**Keywords:** Primary hyperparathyroidism, Intact PTH, Ionised calcium, Hypercalcemia, Parathyroid adenoma

## Abstract

**Background:**

Primary hyperparathyroidism is a common endocrine disorder. Hypercalcemia with normal PTH levels is very unusual and can lead to diagnostic difficulties. There are very few very few studies in the literature and all with limited numerical samples. The goal of the present study was to determine the real incidence and characteristics of primary hyperparathyroidism with normal PTH and to evaluate if intraoperative PTH testing is useful in these patients.

**Methods:**

We performed a retrospective review of 314 patients who had undergone parathyroidectomy to treat primary hyperparathyroidism between January 2002 and December 2016. Patients were divided in two groups according to biochemical preoperative findings: in Group A were included patients with normal serum PTH, in Group B those with increased serum PTH.

**Results:**

Nine patients (3.7%) were included in group A and 235 in group B. Patients in group A were younger (51.5 ± 12.9 years vs 59.6 ± 12.5); preoperative serum calcium and the incidence of coexisting thyroid disease were similar between the two groups. Symptomatic patients were more frequent in Group A (77.8% vs 39.1%; *p* = 0.048). There were no significant differences regarding preoperative localization studies and surgical procedure. Intraoperative PTH determination demonstrated sensitivity of 86% in group A and 97% in group B, specificity and positive predictive value of 100% in both the groups, negative predictive value of 67% in group A and 79% in group B. Histopathological examination demonstrated a single gland disease in 8 (88.9%) patients in group A and a multi gland disease in 1 (11.1%), in group B single gland disease was found in 218 (92.8%) patients and multi gland disease in 17 (7.2%). Unsuccessful surgery with persistent or recurrent hyperparathyroidism occurred in 1 (11.1%) patient in group A and 4 (1.7%) in group B.

**Conclusions:**

Primary hyperparathyroidism with normal PTH is rare but physicians should be aware of this possibility in patients with hypercalcaemia. Patients with normal PTH levels are younger and more frequently symptomatic. Intraoperative PTH testing plays an important role in the operative management even in such patients.

## Background

Primary hyperparathyroidism (PHP) is the third most common endocrine disorder in the United States and the second most common cause of hypercalcemia [1–7].

The diagnosis of PHP is classically based on the finding of hypercalcemia in the presence of high (or non suppressed) PTH levels [6, 8–11]; additional laboratory hallmark features are hypophosphatemia and elevated urinary cyclic adenosine monophospate [10]. Unfortunately, the diagnosis is not always so simple and a number of other factors must be considered [6]. Preoperative serum calcium and intact-parathyroid hormone (iPTH) levels are the most useful diagnostic parameters that allow differentiating PHP from non-parathyroid-dependent hypercalcemia [10]. Parathyroid adenomas, predominantly composed of chief cells, are the most common cause of PHP [10].

While normocalcemic hyperparathyroidism is well recognized in PHP, less is known about patients with high calcium but normal iPTH possibly leading to diagnostic difficulties [1, 3, 9, 10, 12–14]. The literature on this condition is poor and consists mainly of clinical cases [3, 4, 12–16]. An incidence of 5 to 33% has been documented in the literature [2, 10, 12, 13, 17] but no etiologic explanation has been given [10]. In 1987, parathyroid-hormone-related peptide (PTHrp) has been isolated as a causative factor of malignant hypercalcemia [10]. Previous studies have suggested that this condition is a less symptomatic, lighter and weaker form of PHP [18].

Intraoperative PTH (IoPTH) testing has revolutionized the way we treat patients with PHP by allowing focused and minimally invasive procedures [1, 19, 20]. The successful removal of hyperfunctioning parathyroid glands is indicated by a decrease in PTH levels > 50% within 10–20 min [1, 21, 22].

The goal of the present study was to determine the real incidence and characteristics of PHP with normal PTH and to evaluate if IoPTH testing is useful in these patients.

## Methods

After approval from our Institutional Board Committee we performed a retrospective review of 314 patients who had undergone parathyroidectomy to treat PHP between January 2002 and December 2016 within the Unit of General and Endocrine Surgery at the University of Cagliari, Italy. Only patients with preoperative PHP diagnosis were included in this study: 61 patients in which diagnosis was made during neck surgery for other diseases as well as 10 patients with persistent or recurrent PHP were excluded from the study.

We analysed the following data: age, sex, comorbidities, coexisting thyroid diseases, serum calcium and PTH levels, preoperative localization studies, surgical procedure, histopathological features and follow up.

Serum PTH level was defined normal for values ranging from 15 pg/ml to 65 pg/ml. Patients were considered symptomatic if presenting with typical symptoms of hyperparathyroidism (urolithiasis, osteoporosis with bone or joint pain, mood or neurological disorders, cardiovascular disease). Preoperative localization studies (neck ultrasound (US) and MIBI scans) were performed preoperatively in all patients.

All the patients were submitted to parathyroidectomy. IoPTH determination was routinely used during surgery to confirm removal of all pathological glands; we defined a positive test when PTH value 10′ after excision of suspected pathological gland was 50% or lesser than preoperative value and when it was within range values (10–65 pg/ml). In case of negative test another measurement was made 20′ after excision and, if negative result was confirmed, a bilateral exploration was performed.

Patient follow-up was performed by serum calcium and PTH dosing once daily during hospitalization, then once a week for the first month and every 6 months thereafter. Definition of persistent or recurrent hyperparathyroidism was used in the case of high serum and calcium PTH levels detected within or after 6 months postoperatively, respectively.

Patients were divided in two groups according to biochemical preoperative findings: in Group A were included patients with PHP and normal serum PTH, in Group B those with PHP and increased serum PTH.

### Statistical analysis

Chi-squared test was used for categorical data and t-test for continuous variables. Results were considered statistically significant if *p* value was ≤0.05. Continuous data are reported as the mean value ± standard error of the mean. Calculations were performed with MedCalc ® 12.7.0.0.

## Results

Two-hundred and forty-four patients with preoperative diagnosis of PHP were included in the study. Nine patients (3.7%) were included in group A (PHP with normal serum PTH), with a mean preoperative serum PTH of 55 ± 12.2 pg/ml, and 235 in group B (PHP with increased serum PTH), with a mean preoperative serum PTH of 305 ± 301.1 pg/ml.

Full demographic and clinical data are reported in Table 1. Patients in group A were younger (51.5 ± 12.9 years vs 59.6 ± 12.5), even if this difference was not statistically significant (*p* = 0.06). Preoperative serum calcium was similar between the two groups (11.2 ± 0.6 mg/dl vs 11.4 ± 1.2 mg/dl; *p* = 0.64); also the incidence of coexisting thyroid disease was similar (33.3% vs 30.6%; *p* = 0.84). Symptomatic patients were more frequent in Group A (77.8% vs 39.1%; *p* = 0.048).

**Table 1:** Demographic and clinical data, preoperative studies

	Group A (*n* = 9)	Group B (*n* = 235)	*p* value
Male	2 (22.2%)	34 (14.4%)	0.86
Female	7 (77.8%)	201 (85.6%)	
Age (years)	51.5 ± 12.9	59.6 ± 12.5	0.06
Preoperative calcium (mg/dl)	11.2 ± 0.6	11.4 ± 1.2	0.64
Preoperative PTH (pg/ml)	55 ± 12.2	305 ± 301.1	0.013
Coexisting thyroid disease	3 (33.3%)	72 (30.6%)	0.84
Symptomatic PHP	7 (77.8%)	92 (39.1%)	0.048
- Cardiovascular disease	4 (22.2%)	41 (17.4%)	0.11
- Nephrolithiasis	2 (22.2%)	24 (10.2%)	0.55
- Osteoporosis	3 (33.3%)	22 (9.4%)	0.07
- Other	2 (22.2%)	25 (10.6%)	0.58
Preoperative studies			
- Concordant MIBI and US	6 (66.7%)	130* (64%)	0.84
- Discordant MIBI and US	3 (33.3%)	73* (36%)	

US: ultrasound; MIBI: ^99m^Tc-Sestamibi scan. * On 203 patients who underwent preoperative both US and MIBI scan

Surgical procedure, histopathological diagnosis and surgical outcome are reported in Table 2. Surgical procedure consisted in mini-invasive parathyroidectomy in 4 (44.4%) patients in group A and in 143 (60.8%) patients in group B. Histopathological examination demonstrated a single gland disease in 8 (88.9%) patients in group A and a multi gland disease in 1 (11.1%); in group B, single gland disease was found in 218 (92.8%) patients and multi gland disease in 17 (7.2%) (*p* = 0.83). Unsuccessful surgery with persistent or recurrent PHP occurred in 1 (11.1%) patient in group A and 4 (1.7%) in group B (*p* = 0.44).

**Table 2:** Surgical procedure, histopathological diagnosis and surgical outcome

	Group A (*n* = 9)	Group B (*n* = 235)	*p* value
MIP	4 (44.4%)	143 (60.8%)	0.52
Bilateral neck exploration	5 (55.6%)	92 (39.2%)	
Operative time (minutes)	91.7 ± 41.3	89.8 ± 38.3	0.88
Postoperative stay (days)	2.7 ± 1.7	2.4 ± 0.7	0.31
Single adenoma	8 (88.9%)	209 (88.9%)	0.59
Double adenoma	1 (11.1%)	6 (2.6%)	0.62
Multigland hyperplasia	0	11 (4.7%)	0.87
Carcinoma	0	9 (3.8%)	0.76
Multi gland disease	1 (11.1%)	17 (7.2%)	0.83
Surgical failure (persistent / recurrent PHP)	1 (11.1%)	4 (1.7%)	0.44

*MIP* Mini invasive parathyroidectomy

IoPTH determination demonstrated sensitivity of 86% in group A and 97% in group B, specificity and positive predictive value of 100% in both the groups, negative predictive value of 67% in group A and 79% in group B (Table 3).

**Table 3:** Intraoperative PTH test results

	Group A (*n* = 9)	Group B (*n* = 235)	*p* value
TP	6 (66.7%)	207 (88.1%)	0.12
TN	2 (22.2%)	22 (9.4%)	
FN	1 (11.1%)	6 (2.6%)	
Sensitivity	86%	97%	
Specificity	100%	100%	
PPV	100%	100%	
NPV	67%	79%	

*TP* True positive result, *TN* True negative result, *FN* false negative result, *PPV* Positive predictive value, *NPV* Negative predictive value

Odds ratio of patients of patients with PHP with normal serum PTH are reported in Table 4.

**Table 4:** Odds ratio of patients with PHP and normal serum PTH

	Odds ratio	95% CI	Z statistic
Symptomatic PHP	6.1630	1.2541–30.2883	2.239
Discordant preoperative studies	0.8904	0.2162–3.663	0.161
Surgical failure	7.2187	0.7224–72.1394	1.683
Multi gland disease	1.6029	0.1892–13.5789	0.433
False Intraoperative PTH test result	4.7708	0.5123–44.4272	1.373

## Discussion

Normohormonal PHP is a distinct PHP entity that exhibits normal PTH levels [14]. Since 1976, approximately 140 cases of hypercalcemia and normal PTH levels due to parathyroid adenoma have been reported in the literature [4, 10, 15, 16]. The true incidence of normohormonal PHP is very difficult to ascertain [14]. The reported incidence in the literature is 3–10.5% [6, 13, 14, 18, 23, 24]. To our knowledge, the cohort of Amin [18] of 58 sporadic PHP patients with normal PTH levels is the largest reported in the literature to date. However, because of the difficulty in recognizing this entity, the number of patients with normohormonal PHP could be much higher [14]. We report 9 cases of PHP with normal PTH with a rate of 3.7%, in line with other reports in the literature.

The first authors to propose potential etiopathogenetic mechanisms were Hollenberg and Arnold [15] in 1991. Theories have included the pulsatile secretion of PTH, the secretion of abnormal PTH altering its measurement but not its function, the presence of unmeasured active PTH fragments, the presence of a circulating antibodies interfering with the assay, the presence of another mediator of hypercalcemia (PTH-related peptide), and the increased peripheral tissue sensitivity to normal PTH [2, 8, 10, 14, 16, 17, 25]. Potential mechanisms to consider are also discrepancies between different types of equipment, methods of dosing and sampling as well as early diagnosis [14]. Wallace [14] also hypothesize lower PTH setpoints in some patients and anatomic barriers in local circulation around a parathyroid neoplasm.

Patients with normal PTH were a decade younger with an increased frequency of premenopausal females compared to the patients with overt PHP [12]. Our experience confirms that patients with normal PTH tend to be younger (51.5 ± 12.9 vs 59.6 ± 12.5 years) even if the data does not reach full statistical significance (*p* = 0.06).

The patients in the normal PTH group did not differ in symptoms and signs from the patients in the overt PHP group, supporting the concept that the PTH level does not correlate with patient symptoms [12, 13, 18, 23]. The normal serum PTH values delayed diagnosis in a significant proportion of patients (40%) in the study of Mischis-Troussard [13]. In our experience patients with normal PTH were more frequently symptomatic (77.7% vs 39.1%, *p* = 0.048) probably in relation to the fact that the diagnosis was taken into consideration later and that, in these cases, we tend to operate only patients with relevant symptomatology.

Patients with normal levels of PTH had smaller adenomas with low bone turn-over and preserved bone density and glomerular filtration rate compared with the patients with overt PHP [12, 13, 18]. In the experience of Amin [18], imaging modalities were less sensitive perhaps for the small gland weight, although the results were not statistically significant. In our experience the sensitivities of US and 99mTc sestamibi parathyroid scintigraphy were comparable to those observed in the entire PHP group; these results were similar to other reports in the literature [13].

Alhefdhi [1] reports higher rates of multi gland diseases; on the contrary, Amin [18] did not find differences. Our results are in line with those of Amin [18]: we detected only one case of multi gland disease among patients with normal PTH and the difference with group B was not statistically significant.

In the experience of Mischis-Troussard [13], the frequency of parathyroid hyperplasia was high (20%), but not enough to conclude that the proportion of hyperplasia is higher in PHP with normal serum PTH levels. Our results are in line with this report.

The use of IoPTH associated with MIP has allowed to reduce the operative time, the length of hospital stay and the costs, as well as to eliminate the risk of bilateral recurrent laryngeal nerve palsies and hypocalcemia [1, 26–28]. In the experience of Amin [18] a MIP or unilateral exploration was feasible in 43% of patients, while most were submitted to a bilateral neck exploration, probably for the smaller gland weight and the problems in preoperative localization [18]. In our experience, we have carried out the same surgical strategy in both groups and the incidence of MIP and bilateral exploration have been similar.

IoPTH is useful to confirm the removal of pathological parathyroids in this group of patients [1]. In our experience, sensitivity and negative predictive value were slightly lower in group A (86% vs 97 and 67% vs 79% respectively), while specificity and positive predictive value were 100% in both the groups, confirming the high reliability of the technique.

Our study is limited by the small number of patients and the retrospective nature of the study. Further multicenter studies with a large number of patients could give more precise indications regarding this pathology for the future.

## Conclusions

PHP with normal PTH is rare but physicians should be aware of this possibility in patients with hypercalcemia. Patients with normal PTH levels are younger and more frequently symptomatic. IoPTH testing plays an important role in the operative management even in such patients.

## Abbreviations

IoPTH: Intraoperative PTH
iPTH: Intact-parathyroid hormone
PHP: Primary hyperparathyroidism
PTH: Parathyroid hormone
PTHrp: Parathyroid-hormone-related peptide
US: Neck ultrasound
